# Intermittent Theta-Burst Stimulation Over the DorsoLateral PreFrontal Cortex (DLPFC) in Healthy Subjects Produces No Cumulative Effect on Cortical Excitability

**DOI:** 10.3389/fpsyt.2021.626479

**Published:** 2021-02-18

**Authors:** Noomane Bouaziz, Charles Laidi, Fanny Thomas, Palmyre Schenin-King Andrianisaina, Virginie Moulier, Dominique Januel

**Affiliations:** ^1^Unité de recherche clinique, Pôle 93G03, EPS de Ville Evrard, Neuilly sur Marne, France; ^2^Pôle de Psychiatrie, Assistance Publique-Hôpitaux de Paris, Faculté de Médecine de Créteil, DMU IMPACT, Hôpitaux Universitaires Mondor, Créteil, France; ^3^Service hospitalo-universitaire de psychiatrie adulte, CH du Rouvray, Sotteville-lès-Rouen, France

**Keywords:** neuro modulation, iTBS, rTMS (repetitive transcranial magnetic stimulation), cortical excitability transcranial magnetic stimulation, neuroexcitability, depression

## Abstract

**Background:** Intermittent Theta Burst Stimulation (iTBS) is a design of repetitive Transcranial Magnetic Stimulation (rTMS) and could be a candidate to replace rTMS in the treatment of depression, thanks to its efficacy, shorter duration, and ease of use. The antidepressant mechanism of iTBS, and whether this mechanism is mediated by a modulation of cortical excitability, remains unknown.

**Methods:** Using a randomized double-blind, sham-controlled trial, 30 healthy volunteers received either iTBS or a sham treatment targeting the left DorsoLateral PreFrontal Cortex (L-DLPFC), twice a day over 5 consecutive days. Cortical excitability was measured before and after the 5 days of stimulation.

**Results:** No difference in cortical excitability was observed between active or sham iTBS.

**Conclusion:** Our study does not support any effect on cortical excitability of repetitive iTBS targeting the L-DLPFC.

## Introduction

Repeated Transcranial Magnetic Stimulation (rTMS) is a validated non-invasive brain stimulation technique used to treat resistant depression (1).

Intermittent Theta Burst Stimulation (iTBS) is a design of rTMS that uses a very highly modulated frequency to produce a high number of pulses in a shorter time. This increases cortical excitability in a more robust and longer lasting way than rTMS, and in a similar way to High-Frequency (HF) rTMS (2).

Several studies have reported that iTBS is non-inferior to the gold standard 10 Hz rTMS in the treatment of major depression (3, 4). Furthermore, one session of iTBS lasts only 3 min, making it more acceptable to participants than longer lasting protocols such as 10 Hz rTMS, which can last 20–40 min (5).

The exact neurobiological mechanisms of iTBS/rTMS that lead to an antidepressant effect remain unknown.

Some etiologies of depression involve an imbalance of inter-hemispheric neuroexcitability. This imbalance has been identified in the dorsolateral prefrontal cortex (DLPFC) via imaging and EEG studies. This has justified the use of high-frequency (HF) rTMS targeting the left DLPFC to treat major depressive episodes (6, 7). Other studies have identified this imbalance as hypoexcitability of the left motor cortex. Some studies relate this hypoexcitability to an increase in the left rMT in patients with depression compared to healthy subjects (7–9). Other studies report that in patients with depression, the rMT of the left motor cortex was higher than that of the contralateral motor cortex (9, 10). In 2006, Bajbouj et al. (11) reported that the rMT of the left cortex was higher than that of the right cortex in depressed patients, whereas in healthy subjects this phenomenon was reversed. This suggests that, in patients with depression, there is an overall deficit of any excitability in the left hemisphere (7). This possibility has also been supported by other authors (12). Nevertheless, as cortical excitability is specifically related to the motor cortex, there is limited evidence that the enhancement of the excitability of the DLPFC using rTMS/iTBS could result in the same effect in the ipsilateral motor cortex.

We have thus stipulated that the remission of depressive symptoms after an increase in DLPFC excitability via HF rTMS (or iTBS) could also lead to a similar increase in the excitability of the ipsilateral motor cortex. A study by Spampinato et al. (13) supports this hypothesis by reporting that the antidepressant efficacy of HF rTMS (10 HZ) targeting the ipsilateral DLPFC was associated with a decrease in the ipsilateral rMT. The same authors hypothesized that an overall pro-excitability effect of HF rtMS would be mediated by the Long-Term Potentiation (LTP) phenomenon. Triggs et al. (14) also reported a significant decrease in the left rMT in ten patients with depression after a two-week open-label trial of 20 Hz rTMS (2,000 pulses/session, 20 sessions) over the ipsilateral DLPFC. Both studies showed an increase in M1 excitability after HF rTMS targeting the ipsilateral DLPFC. Based on these studies, we hypothesized that iTBS (having the same pro neuroexcitatory profile) targeting the left DLPFC would lead to an increase in the excitability of the ipsilateral M1.

The aim of this study was to assess the effect of iTBS on cortical excitability in the motor cortex in healthy controls.

## Materials and Methods

This study is a randomized double-blind vs. placebo trial designed to assess cortical excitability in the motor cortex before and after ten sessions of iTBS over the L-DLPFC.

### Participants

Thirty volunteers, who had given written consent, were randomly assigned to either an active or a sham iTBS group. Healthy subjects between 18 and 65 years old, with no known history of psychiatric illness, and who had been matched in age, gender and education level, were included. They had to be right-handed, with a BDI (Beck Depression Inventory) score of <8, and an HDRS (Hamilton Depression Rating Scale) score of <8. Healthy female subjects had to confirm that they were not pregnant, and were on oral contraception. The non-inclusion criteria consisted of: being on any psychotropic drug, having any psychiatric disorder on axis I or II of the DSM-IV-TR (Diagnostic and Statistical Manual of Mental Disorders: Revised text), suffering from epilepsy, or having any other contraindications for rTMS.

All participants gave written consent, and the study was approved by the local ethical committee (CPP Ile de France VIII, number 101078, ID-RCB 2010A01032-37).

### Cortical Excitability Assessment Procedures

Surface electromyograms were taken from abductor pollicis brevis muscles (APB) via electromyographic (EMG) self-adhesive electrodes, with solid gel coated disposable Ag/AgCl electrodes placed on the belly and tendons of the APB muscles.

To assess cortical excitability, TMS was performed using a standard 70 mm figure-of-eight coil (MCF-B65 Butterfly Coil) attached to a Medtronic MagOption stimulator. The coil was attached to the head with the handle pointing backwards and laterally, at an angle of 45° to the sagittal plane.

EMG activity was amplified 10,000-fold using a Matrix Light amplifier (Micromed, Mâcon, France) through filters set at 20 Hz and 2 kHz with a sampling rate of 16 kHz. The activity was then recorded by a computer using SystemPlus EVOLUTION software (version 1.04, Micromed, Mâcon, France).

### Cortical Excitability Parameters Assessment Paradigm

The cortical excitability of each subject was assessed at baseline and 48 h after the end of the treatment. The following features of the left and right cortices were assessed: resting motor threshold (rMT), motor evoked potentials (MEPs), and intracortical facilitation (ICF).

The rMT was defined as the lowest TMS intensity required to induce an MEP with an amplitude of at least 50 microVolts (μV) in at least five out of ten trials (15).

Motor evoked potentials were defined as the overall reaction of a peripheral muscle, as measured by electromyography (EMG), that were induced via TMS of the contralateral motor cortex (16). The peak-to-peak amplitude of MEPs produced from a single TMS pulse provides an objective measure of corticospinal excitability (16). Our standard MEP or “test stimulus” was obtained after a single pulse delivered at 120% of the rMT.

Intracortical facilitation (ICF) was performed using two stimuli with an inter-stimulus interval (ISI) of 15 ms (melliseconds) Intracortical facilitation is believed to be mediated by excitatory inputs from glutamatergic pathways (17). In our study, the intensity of the test pulse was at a suprathreshold of 120% of the rMT and the intensity of the conditioned pulse was at a subthreshold of 80% of the rMT.

For all these excitability parameters, five trials were recorded with an interval of at least 5 s between each trial. For the rMT and MEPs, results were expressed using the mean and standard deviation of the five trials. For the ICF, results were expressed as the ratio of the average of the five MEPs obtained via the ICF paradigm to the average of the standard MEPs (13).

### iTBS Procedure

iTBS was applied using parameters described by Huang et al. (2), except for the intensity of the stimulation, which was at 80% of the rMT in our study compared to 80% of the active motor threshold (aMT) initially used by Huang and colleagues in 2005 (2).

Using an intensity at 80% of the rMT instead of 80% of the active motor threshold (aMT) allows for higher stimulation power. Two of the most cited studies that have used iTBS in the treatment of resistant depression, namely Blumberger et al. (4) and Cole et al. (18) used stimulation intensity at 120 and 90% of the rMT respectively, and reported very good tolerance. The use of the rMT instead of aMT is also methodologically easier, since TMS interferes with the ability to maintain steady muscle contraction with stable background EMG activity of 10–20% of maximal contraction (6). Furthermore, the use of aMT could result in the spontaneous pre-activation of target muscles, which may alter the aftereffects of iTBS (18).

Three 50 Hz stimuli were repeated at a frequency of 200 ms for 2 s, which constituted a stimulation train. This train was then repeated every 10 s with a total stimulation duration of 190 s, and a total number of stimuli at 600.

A Magstim Super Rapid stimulator (Magstim, Wales) was used with a 70 mm double air film coil. For the control group, a sham coil providing the same acoustic sensation and visual impact as the active coil was used. The sham coil stimulated the skin and subcutaneous tissue of the scalp, giving the subjects the sensation of magnetic stimulation. The coil was positioned over the left DLPFC using Brainsight, an MRI-guided neuronavigation software (Rogue Research Inc., Canada).

MRI scans were performed on a 3 Tesla scanner (Siemens Healthcare, Erlangen, Germany). A 3D T1-weighted sequence was acquired with the following parameters: 176 contiguous slices, slice thickness = 1.0 mm, repetition time (TR) = 2,300 ms, echo time (TE) = 2.98 ms, field of view = 256^*^256.

The rTMS coil was applied over the L-DLPFC, which was targeted using established spatial coordinates for this area (Montreal Neurological Institute (MNI) coordinates: x = −50, y = 30, z = 36) (19). To do this, one spherical region of interest (ROI) of a 5 mm radius and centered at these MNI coordinates was generated using the MarsBaR toolbox (http://marsbar.sourceforge.net/) (20). The ROI was de-normalized from the MNI space to the individual space on each patient using the inverse transformation obtained from the VBM8 toolbox. The rTMS coil was positioned tangential to the scalp location overlying the L-DLPFC using an MRI-based frameless stereotactic neuronavigation system (Brainsight, Rogue Research Inc., Montreal, Canada).

### Statistical Analysis

Differences between subjects in active and sham arms for sociodemographic characteristics were compared with a Student's *t*-test.

We computed the delta (baseline - after stimulation) for each measurement (rMT, MEPs, ICF) and for each group (active or sham).

We compared the delta in the active and sham group.

The Gaussian Distribution of each variable was evaluated by the Shapiro-Wilk test.

Some of the variables did not meet the assumptions for parametric statistics (Gaussian distribution for t-test, normal distribution for the residuals of the ANOVA) and is why we chose non-parametric statistical tests (Mann–Whitney U) for some. For the normally distributed variables, we performed t-test instead of ANCOVA because our groups were matched for age and gender, thus there was no confound that we could take into account. All statistical analyses were conducted using Python Statsmodels, an open-source library (21).

## Results

There were no significant differences in age or gender ratio between active and placebo groups at baseline (Table 1).

**Table 1:** Sociodemographic characteristics in iTBS and sham groups.

**Variables**	**Active iTBS arm**	**Sham iTBS arm**	***p* value**
Number	14	16	
Age (mean, SD)	24.57 (6.65)	25.75 (6.19)	0.61
Gender ratio (M/F)	1	1	-
Handedness (mean, SD)	87 (9.66)	87 (15)	1
Level of education (mean, SD)	15.35 (1.69)	14.5 (1.63)	0.16

*SD, standard deviation; M, male; F, female; iTBS, intermittent theta burst stimulation*.

**Figure 1:**
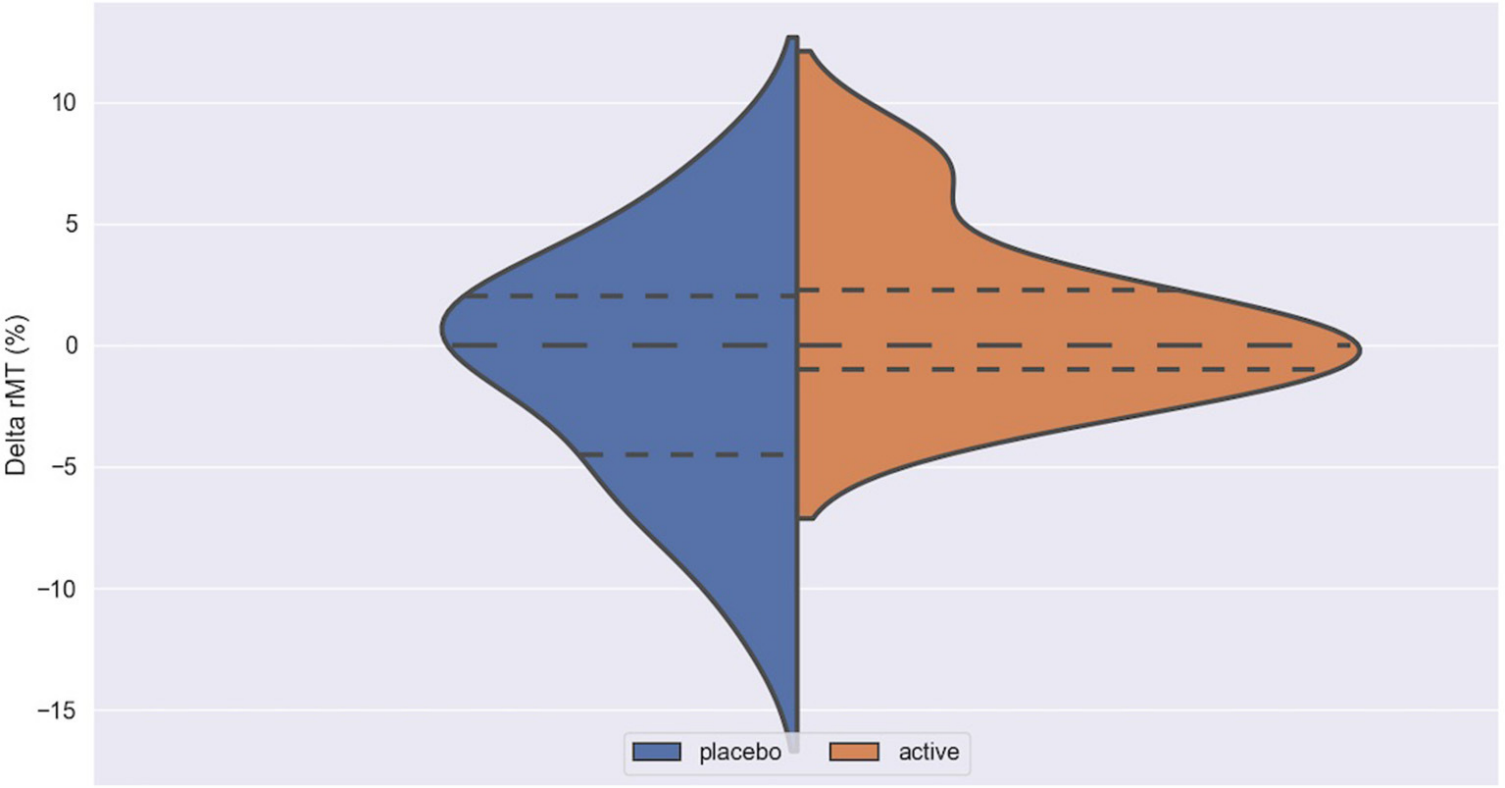
Delta rMT in active and placebo groups in left cortex. rMT, resting motor threshold; Delta rMT, difference in rMT before and after iTBS.

**Figure 2:**
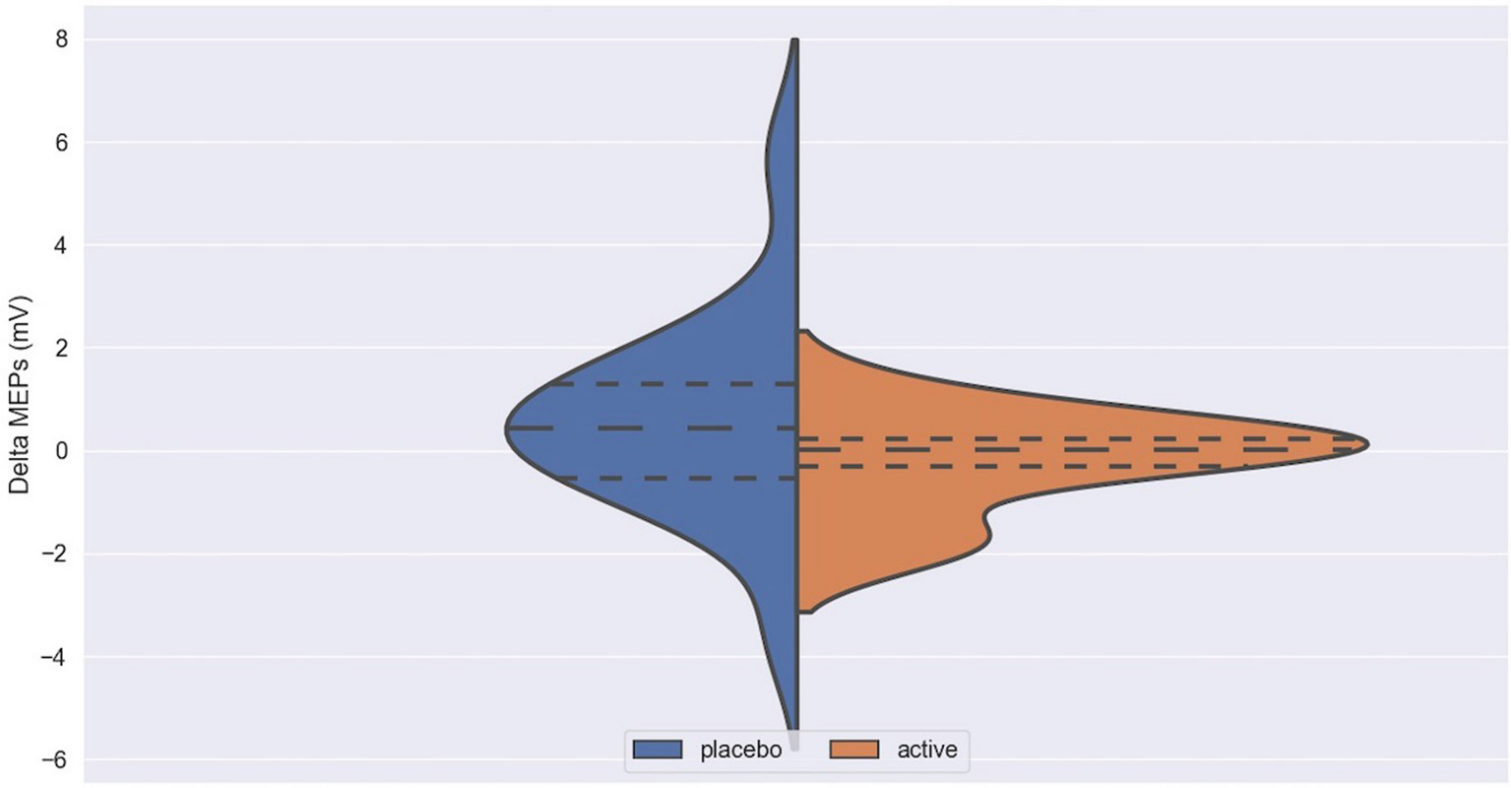
Delta MEPs in active and placebo groups in left cortex. MEPs, Motor Evoked Potentials at 120% of rMT; Delta MEPs, difference in MEPs before and after iTBS.

**Figure 3:**
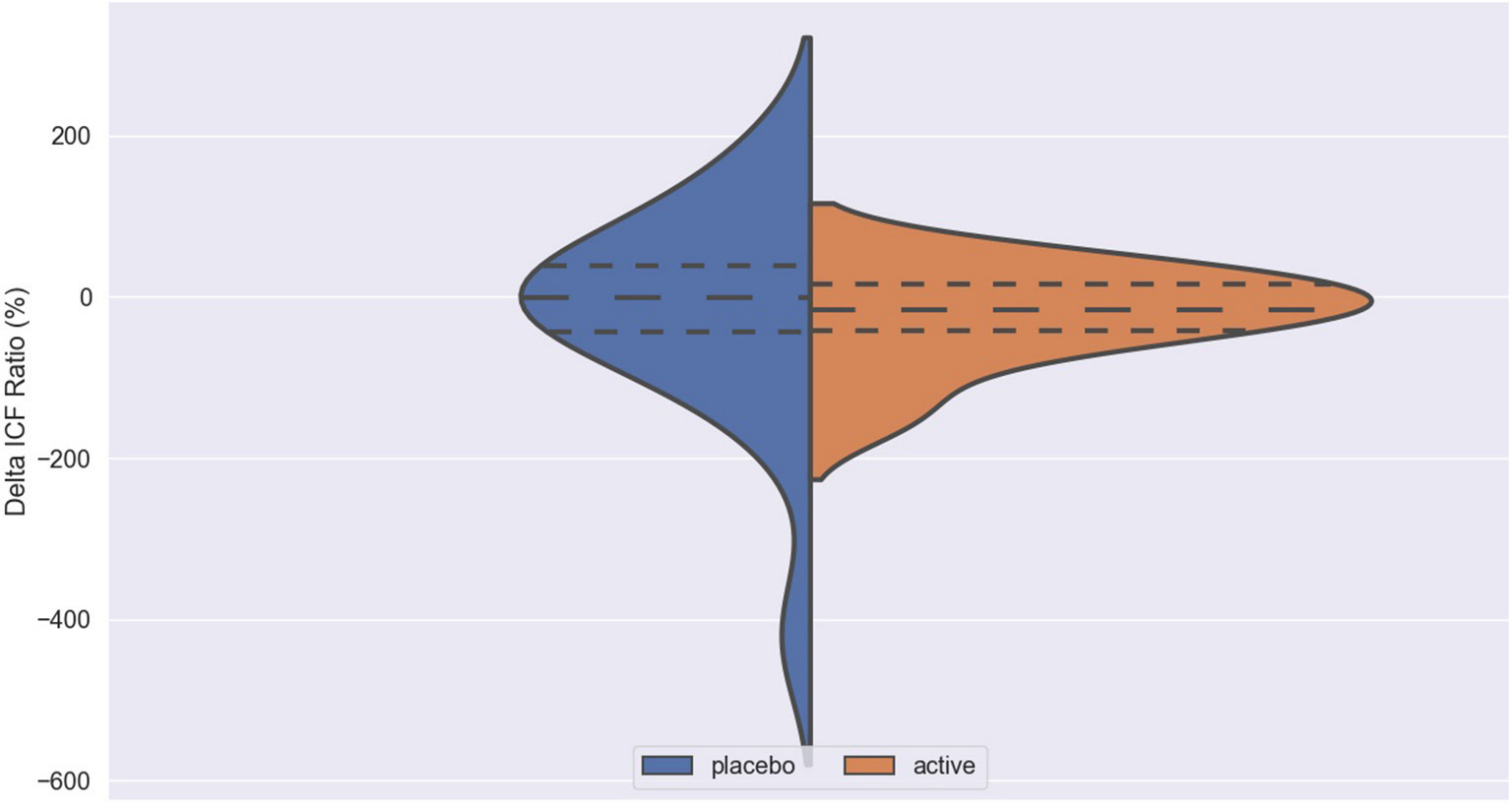
Delta ICF in active and placebo groups in left cortex. ICF, intracortical facilitation; Delta ICF, difference in ICF before and after iTBS. Delta: difference in the resting Motor Threshold (rMT, Figure 1) after minus before active or sham stimulation, the Motor Evoked Potentials (MEPs, Figure 2) and Intracortical Facilitation (ICF) before and after intermittent Theta Burst Stimulation (iTBS) in the placebo (blue) and the active (orange) arm. Hashed lines delineate quartile of the distribution; to central hashed lines refer to the mean of the distribution. We choose the violin plots to provide information about the distribution of the variables, which is not possible with box plots and/or scatter plots. We believe that this representation of the results gives additional information to the reader. The x axis represents both the placebo (blue) and active (orange) group.

The impact of iTBS and sham stimulation on all endpoint parameters is reported in Table 2 and in Figures 1–3.

**Table 2:** Difference (before and after stimulation) in the placebo and the active arm in resting motor threshold, motor evoked potentials and intracortical facilitation of both cortices before and after stimulation.

**Excitability parameters**	**Target**	**Active baseline**	**Active post iTBS**	**Active delta**	**Sham baseline**	**Sham post iTBS**	**sham delta**	**Comparison between active delta and sham delta**	***p-*value**
rMT^**^ Mean (sd)	R DLPFC	52.15 (6.96)	52.81 (8.49)	0.63 (2.97)	51.4 (8.39)	51.93 (7.79)	0.5 (4.5)	MW stat. = 79.5	0.44
	L DLPFC	53.61 (7.8)	55.08 (8.83)	1.16 (3.37)	51.33 (9.18)	50.46 (8.31)	−0.86 (4.88)	*T*-test stat. = 1.22	0.23
MEP^*^ Mean (sd)	R DLPFC	1.51 (1.16)	1.26 (1.06)	−0.25 (1.24)	1.43 (0.95)	1.59 (1.29)	0.09 (1.42)	*T*-test stat. = 0.68	0.5
	L DLPFC	1.86 (1.03)	1.64 (1.05)	−0.22 (0.94)	1.71 (1.22)	2.30 (2.25)	0.58 (1.96)	*T*-test stat. = 1.34	0.18
Ratio ICF^*^ Mean (sd)	R DLPFC	121.21 (72.41)	131.17 (87.49)	7 (116)	136.88 (133.17)	201.20 (195.25)	72.53 (250)	*T*-test stat. = 1.22	0.23
	L DLPFC	125 (58)	102.64 (39)	−23.25 (136.69)	169.94 (181)	151.92 (106.47)	−23 (58)	MW stat. = 94	0.33

** *2 missing values*;

* *one missing value, rMT, resting motor threshold (% of maximal output); MEP, motor evoked potential (120% of rMT in μV); Ratio ICF, Intracortical facilitation ration; t-test, the Student's t-test; MWt, Mann–Whitney U test; R-DLPFC, right dorsolateral prefrontal cortex; L-DLPFC, left dorsolateral prefrontal cortex; SD, standard deviation*.

No difference was found between active and placebo stimulation in the rMT, MEPs and ICF in the right and the left cortex. There was also no trend suggesting a difference between the active and the sham arm.

## Discussion

We found no difference in all cortical excitability measurements between active and placebo stimulation in the left or right hemisphere. This negative result was contrary to our expectations.

The DLPFC has various anatomical projections on M1. These two regions are believed to work closely together to form fundamental circuitry involved in motor tasks of varying complexity (22). It is also widely believed that the impact of rTMS occurs if sessions of rTMS are repeated over a period of several days, leading to a “build-up” effect (23).

We therefore hypothesized that repeated sessions of iTBS over the L-DLPFC would result in increased excitability of the ipsilateral M1.

Studies assessing iTBS on cortical excitability are scarce, but can be divided into three types: (i) studies exploring TBS impact on cortical excitability after only one session of stimulation targeting the M1, (ii) studies exploring iTBS impact on cortical excitability after repeated sessions targeting the M1, (iii) studies exploring iTBS impact on cortical excitability targeting the DLPFC (a protocol template for treating depression).

Regarding (i), Chung et al. (16) replicated the findings of Huang et al. (2), being that one session of iTBS over the M1 in healthy subjects results in an increase in excitability by enhancing MEP amplitude, and that continuous Theta Burst Stimulation (cTBS) results in the opposite effect.

Regarding (ii), Perellón-Alfonso et al. (24) reported that over a 5-day “treatment” protocol, cumulative active iTBS over the M1 in 20 healthy volunteers was not superior to sham stimulation at inducing long-lasting facilitation of corticospinal excitability.

To date (iii), only Cao et al. (25) has assessed the effects of iTBS over the DLPFC on M1 cortical excitability. These authors reported that one iTBS session over the DLPFC, in 15 healthy right-handed participants, decreased the MEP amplitude in the M1, whereas the cTBS session resulted in the opposite effect. The authors explained their results by the Zero-Sum Enhancement theory. According to this theory, the increase in the excitability of the DLPFC is necessarily accompanied by a decrease in the excitability of the M1 at the other end of the network (26).

Triggs et al. (14) and Spampinato et al. (13) reported the opposite results after several sessions of HF rTMS (thought to have the same excitatory properties as iTBS) over the L-DLPFC. They reported a decrease in the rMT of the ipsilateral motor cortex.

Brown et al. (22) found no effect on M1 excitability after stimulation of the ipsilateral DLPFC using the TMS Conditioning-Test Approach. This is a method to evaluate the impact of a TMS conditioning stimulus (CS) applied over the frontal cortex on MEPs elicited by the TMS test stimulus (TS) over the M1 (22).

Our study is the first to evaluate the effect of iTBS over the L-DLPFC on cumulative cortical excitability in healthy subjects, using a “depression stimulation protocol.” Our results do not support any impact of repeated iTBS sessions applied over the L-DLPFC on the excitability of the M1.

The result of our study, especially in view of the scarcity of studies on the subject and their contradictory results, is difficult to explain. Nevertheless, some hypotheses are possible.

First, although a DLPFC-M1 connection has been widely reported (22, 25, 26), it may not be direct, and may be influenced by postsynaptic connections with other brain regions (basal ganglia, thalamus) (22).

Secondly, the stimulated target and coil orientation were probably not optimal to test our hypothesis. Indeed, we targeted an area traditionally used for the treatment of depression, whereas it could have been preferable to vary the target area from patient to patient, to identify a specific area more likely to induce excitability in M1.

Thirdly, the hypothesis linking the antidepressant effect of rTMS/iTBS to a change in cortical excitability may not be accurate. This is because the antidepressant action may be mediated by other neurobiological mechanisms that are distinct from a simple modulation of cortical excitability, such as modulation in dopamine and/or glutamate, connectivity between non-motor brain regions, modulation of gene expression, de novo expression of proteins, morphological changes, and/or changes in intrinsic firing patterns of diverse neocortical neurons or intrinsic membrane properties (27).

The conclusion of our study, which does not support any impact of repeated iTBS sessions applied over L-DLPFC on the excitability of the M1 should be taken with caution. Despite its original design, our study suffers from several limitations: first, the relatively small sample size may have resulted in Type 2 errors. Secondly, our methodology for collecting cortical excitability parameters is probably not optimal. In fact, we conducted five trials of each variable, but more trials would be preferable. Using Transcranial Magnetic Stimulation (TMS) to assess cortical excitability (single pulse TMS for corticospinal processes, rMT and MEPs and paired pulses for intracortical processes) can be considerably affected by interpersonal, intrapersonal and intersessional variability, which reduces the sensitivity and reproducibility of this method. Our results showed a significant interpersonal and intrapersonal variability in cortical excitability between the different sessions that was partly due to the choice of conducting an average of only five trials for each parameter. Biabani et al. (28) reported that the optimal number of TMS trials needed for reproducible measurements of corticospinal excitability and intracortical inhibition was 26. Conducting 30 trials resulted in a significant improvement in the reproducibility of ICF in a single session, but only moderately improved the reproducibility between different sessions. Goldsworthy et al. (29) reported that 20–30 TMS trials are optimal to ensure a stable measure of MEP amplitude with high within- and between-session reliability. Chang et al. (30) reported that the optimal number of neuronavigated trials required to improve the reliability of the evaluation of the amplitude of MEP, latency of MEP, ICF and Short-Interval Intracortical Inhibition (SICI), are 21, 23, 20, and 25 respectively. However, these three authors used 5 out of 10 trials to determine the participants' rMT, as we did.

To the best of our knowledge, the majority of studies using TMS or iTBS in physiology or in therapeutic trials use at least five out of ten positive trials (motor response and/or induction of an EMP with amplitude ≥50 μV) to determine the motor threshold. However, the updated IFCN guidelines Rossini et al. (6) suggest using 10 MEPs out of 20 trials to improve the accuracy of these measurements. In view of this, we suggest that despite it being probably time-consuming, an increase in the number of trials, potentially coupled with a neuronavigation of MEPs, to evaluate the different assessments of cortical excitability parameters, might ultimately increase the consistency of this method of investigation. Thirdly, the reassessment of cortical excitability 48 h after the last iTBS session may be too late, as the brain has probably regained its baseline excitability state through the subsequent homeostatic synaptic plasticity phenomena (27). Our choice to evaluate the effect of repeated iTBS sessions on excitability after 48 h after the last stimulation session was motivated by the search for a possible persistent build-up of cumulative effects on neuroplasticity. Indeed, if iTBS draws its potential antidepressant effect from its modulation of cortical excitability, it would be expected that this effect would persist beyond the stimulation sessions alone. Evaluating immediately or close after the last stimulation session may results in the assessment of only the immediate effect of this last session.

Studies evaluating the cumulative effects of iTBS (or rTMS) on cortical excitability are rare.

Bäumer et al. (31) reported that while the effects of rTMS on ICF after 1Hz rTMS remains 30 min, the effect lasts up to 2 h when the stimulation was made on two consecutive days suggesting build up excitatory effect. Indeed, the evaluation of excitability before each session, 30 min after each session, or before the first session, 30 min after the first session and 30 min after the last session, in addition to the 48-h assessment, could have provided us with more information on the effects of iTBS on cortical excitability in the short- and medium- term.

Another limitation is the fact that in our study we did not control the timing of inclusion, stimulation and assessment of excitability in relation to the phase of the menstrual cycle, which could be a limitation of our study. Given the potential influence of female hormones on cortical excitability, one could speculate that rMT and other indices of cortical excitability may vary with different phases of the menstrual cycle. Indeed, and despite heterogeneous studies, female hormones probably impact cortical excitability. Smith et al. (32) described an excitatory neuronal effect associated with estradiol, and an inhibition effect associated with progesterone. Inghilleri et al. (33) reported similar results, with cortical excitability during the follicular period increasing in conjunction with an increase in estrogen levels. Recently, Schloemer et al. (34) reported that the fluctuation in estrogen (but not progesterone) levels modulates cortical excitability in non-motor (somatosensory and visual) cortices. However, Chagas et al. (35) did not find any variation in rMT according to the phases of the menstrual cycle. Nevertheless, they reported an increase in rMT in women with amenorrhea compared to those at the beginning of their menstrual cycle. In this study our female volunteers were only included if they were taking hormonal contraceptives, which may have minimized the possible influence of hormonal variations caused by the menstrual cycle on cortical excitability. Gender differences were not assessed too. Few studies have evaluated the hypothetical difference in cortical excitability between women and men. Cantone et al. (36) who assessed a large Italian cohort, found some differences between males and females in TMS-induced MEPs of the lower limbs, but not of the upper limbs. Either way, data from the studies cited above strongly suggest that the hormonal status of the women participating in the cortical excitability studies is probably a confounding factor, and should be considered in future studies.

Despite these limitations, we believe that our study has strengths, and that its original design would benefit from being replicated and improved.

To the best of our knowledge, this randomized double-blind controlled study is the first to evaluate the effect of iTBS over the L-DLPFC on cortical excitability in healthy subjects, using a “depression stimulation” protocol with repetitive sessions. Furthermore, this is the largest trial assessing TBS impact on cortical excitability in healthy volunteers. Our study corroborates the safety and the good tolerability of repeated sessions of iTBS.

Even with a higher intensity than that initially used by Huang et al. (2) (80% of rMT instead of 80% of aMT), we observed no serious adverse effects: only a few cases of mild headache that did not persist.

## Data Availability Statement

The raw data supporting the conclusions of this article will be made available by the authors, without undue reservation.

## Ethics Statement

The studies involving human participants were reviewed and approved by CPP Ile de France VIII, number 101078, ID-RCB 2010A01032-37. The patients/participants provided their written informed consent to participate in this study. Written informed consent was obtained from the individual(s) for the publication of any potentially identifiable images or data included in this article.

## Author Contributions

NB: wrote the article, contributed to the conception and design of the study, carried the statistical analysis, and contributed to the carrying out of cortical excitability procedures. CL: contributed to the writing of the article and the carrying out of the statistical tests. FT: contributed to the carrying out of cortical excitability procedures. PS-K: contributed to magnetic stimulation, participant monitoring and to the carrying out of cortical excitability procedures. VM: contributed to the design of the study and the methodological follow-up. DJ: contributed to the conception and design of the study, as well as the writing of the article. All authors contributed to the article and approved the submitted version.

## Conflict of Interest

The authors declare that the research was conducted in the absence of any commercial or financial relationships that could be construed as a potential conflict of interest.

## References

[1] Milev RV Giacobbe P Kennedy SH Blumberger DM Daskalakis ZJ Downar J. Canadian network for mood and anxiety treatments (CANMAT) 2016 clinical guidelines for the management of adults with major depressive disorder: section 4. Neurostimulation Treatm Can J Psychiatry. (2016) 61:561–75. 10.1177/0706743716660033 27486154 PMC4994792

[2] Huang Y-Z Edwards MJ Rounis E Bhatia KP Rothwell JC. Theta burst stimulation of the human motor cortex. Neuron. (2005) 45:201–6. 10.1016/j.neuron.2004.12033 15664172

[3] Bakker N Shahab S Giacobbe P Blumberger DM Daskalakis ZJ Kennedy SH. rTMS of the dorsomedial prefrontal cortex for major depression: safety, tolerability, effectiveness, and outcome predictors for 10 Hz versus intermittent theta-burst stimulation. Brain Stimul. (2015) 8:208–15. 10.1016/j.brs.2014.11002 25465290

[4] Blumberger DM Vila-Rodriguez F Thorpe KE Feffer K Noda Y Giacobbe P. Effectiveness of theta burst versus high-frequency repetitive transcranial magnetic stimulation in patients with depression (THREE-D): a randomised non-inferiority trial. Lancet. (2018) 391:1683–92. 10.1016/S0140-6736(18)30295-2 29726344

[5] Suppa A Huang Y-Z Funke K Ridding MC Cheeran B Di Lazzaro V. Ten years of theta burst stimulation in humans: established knowledge, unknowns and prospects. Brain Stimul. (2016) 9:323–35. 10.1016/j.brs.2016.01006 26947241

[6] Rossini PM Burke D Chen R Cohen LG Daskalakis Z Di Iorio R. Non-invasive electrical and magnetic stimulation of the brain, spinal cord, roots and peripheral nerves: Basic principles and procedures for routine clinical and research application. An updated report from an IFCN committee. Clin Neurophysiol. (2015) 126:1071–107. 10.1016/j.clinph.2015.02001 25797650 PMC6350257

[7] Khedr EM Elserogy Y Fawzy M Elnoaman M Galal AM. Global cortical hypoexcitability of the dominant hemisphere in major depressive disorder: a transcranial magnetic stimulation study. Neurophysiol Clin. (2020) 50:175–83. 10.1016/j.neucli.2020.02005 32169427

[8] Lefaucheur JP Lucas B Andraud F Hogrel JY Bellivier F Del Cul A. Inter-hemispheric asymmetry of motor corticospinal excitability in major depression studied by transcranial magnetic stimulation. J Psychiatr Res. (2008) 42:389–98. 10.1016/j.jpsychires.2007.03001 17449060

[9] Concerto C Lanza G Cantone M Pennisi M Giordano D Spampinato C. Different patterns of cortical excitability in major depression and vascular depression: a transcranial magnetic stimulation study. BMC Psychiatry. (2013) 13:300. 10.1186/1471-244X-13-300 24206945 PMC4226249

[10] Maeda F Keenan JP Pascual-Leone A. Interhemispheric asymmetry of motor cortical excitability in major depression as measured by transcranial magnetic stimulation. Br J Psychiatry. (2000) 177:169–73. 10.1192/bjp.177.2169 11026958

[11] Bajbouj M Lisanby SH Lang UE Danker-Hopfe H Heuser I Neu P. Evidence for impaired cortical inhibition in patients with unipolar major depression. Biol Psychiatry. (2006) 59:395–400. 10.1016/j.biopsych.2005.07036 16197927

[12] Kinjo M Wada M Nakajima S Tsugawa S Nakahara T Blumberger DM. Transcranial magnetic stimulation neurophysiology of patients with major depressive disorder: a systematic review and meta-analysis. Psychol Med. (2020) 1–10. 10.1017/S0033291720004729 33267920 PMC7856413

[13] Spampinato C Aguglia E Concerto C Pennisi M Lanza G Bella R. Transcranial magnetic stimulation in the assessment of motor cortex excitability and treatment of drug-resistant major depression. IEEE Trans Neural Syst Rehabil Eng. (2013) 21:391–403. 10.1109/TNSRE.20132256432 23559064

[14] Triggs WJ McCoy KJ Greer R Rossi F Bowers D Kortenkamp S. Effects of left frontal transcranial magnetic stimulation on depressed mood, cognition, and corticomotor threshold. Biol Psychiatry. (1999) 45:1440–6. 10.1016/S0006-3223(99)00031-1 10356626

[15] Bunse T Wobrock T Strube W Padberg F Palm U Falkai P. Motor cortical excitability assessed by transcranial magnetic stimulation in psychiatric disorders: a systematic review. Brain Stimul. (2014) 7:158–69. 10.1016/j.brs.2013.08009 24472621

[16] Chung SW Hill AT Rogasch NC Hoy KE Fitzgerald PB. Use of theta-burst stimulation in changing excitability of motor cortex: a systematic review and meta-analysis. Neurosci Biobehav Rev. (2016) 63:43–64. 10.1016/j.neubiorev.2016.01008 26850210

[17] Radhu N de Jesus DR Ravindran LN Zanjani A Fitzgerald PB Daskalakis ZJ. A meta-analysis of cortical inhibition and excitability using transcranial magnetic stimulation in psychiatric disorders. Clin Neurophysiol. (2013) 124:1309–20. 10.1016/j.clinph.2013.01014 23485366

[18] Cole EJ Stimpson KH Bentzley BS Gulser M Cherian K Tischler C. Stanford accelerated intelligent neuromodulation therapy for treatment-resistant depression. Am J Psychiatry. (2020) 177:716–26. 10.1176/appi.ajp.2019.19070720 32252538

[19] Rusjan PM Barr MS Farzan F Arenovich T Maller JJ Fitzgerald PB. Optimal transcranial magnetic stimulation coil placement for targeting the dorsolateral prefrontal cortex using novel magnetic resonance image-guided neuronavigation. Hum. Brain Mapp. (2010) 31:1643–52. 10.1002/hbm20964 20162598 PMC6871247

[20] Brett M Anton J-L Valabregue R Poline J-B. Region of interest analysis using an SPM toolbox [Abstract]. Neuroimage. (2002). 16. 10.1016/S1053-8119(02)90013-311771970

[21] Seabold S Perktold J. Statsmodels: econometric and statistical modeling with python. In: van der Walt S Millman J, editors. Proceedings of the 9th Python in Science Conference. Austin, TX: Millman; Jarod (2010). p. 92–96. 10.25080/Majora-92bf1922-011

[22] Brown MJN Goldenkoff ER Chen R Gunraj C Vesia M. Using dual-site transcranial magnetic stimulation to probe connectivity between the dorsolateral prefrontal cortex and ipsilateral primary motor cortex in humans. Brain Sci. (2019) 9:177. 10.3390/brainsci9080177 31357468 PMC6721325

[23] Lefaucheur J-P André-Obadia N Antal A Ayache SS Baeken C Benninger DH. Evidence-based guidelines on the therapeutic use of repetitive transcranial magnetic stimulation (rTMS). Clin Neurophysiol. (2014) 125:2150–206. 10.1016/j.clinph.2014.05021 25034472

[24] Perellón-Alfonso R Kralik M Pileckyte I Princic M Bon J Matzhold C. Similar effect of intermittent theta burst and sham stimulation on corticospinal excitability: A 5-day repeated sessions study. Eur J Neurosci. (2018) 48:1990–2000. 10.1111/ejn14077 30022548

[25] Cao N Pi Y Liu K Meng H Wang Y Zhang J. Inhibitory and facilitatory connections from dorsolateral prefrontal to primary motor cortex in healthy humans at rest-An rTMS study. Neurosci Lett. (2018) 687:82–7. 10.1016/j.neulet.2018.09032 30243883

[26] Pascual-Leone A Horvath JC Robertson EM. Enhancement of normal cognitive abilities through noninvasive brain stimulation. In: Chen R Rothwell JC, editors. Cortical Connectivity: Brain Stimulation for Assessing and Modulating Cortical Connectivity and Function. Berlin, Heidelberg: Springer (2012). p. 207–49. 10.1007/978-3-662-45797-9_11

[27] Cirillo G Pino GD Capone F Ranieri F Florio L Todisco V. Neurobiological after-effects of non-invasive brain stimulation. Brain Stimul. (2017) 10:1–18. 10.1016/j.brs.2016.11009 27931886

[28] Biabani M Farrell M Zoghi M Egan G Jaberzadeh S. The minimal number of TMS trials required for the reliable assessment of corticospinal excitability, short interval intracortical inhibition, and intracortical facilitation. Neurosci Lett. (2018) 674:94–100. 10.1016/j.neulet.2018.03026 29551425

[29] Goldsworthy MR Hordacre B Ridding MC. Minimum number of trials required for within- and between-session reliability of TMS measures of corticospinal excitability. Neuroscience. (2016) 320:205–9. 10.1016/j.neuroscience.2016.02012 26872998

[30] Chang WH Fried PJ Saxena S Jannati A Gomes-Osman J Kim Y-H. Optimal number of pulses as outcome measures of neuronavigated transcranial magnetic stimulation. Clin Neurophysiol. (2016) 127:2892–7. 10.1016/j.clinph.2016.04001 27156431 PMC4956494

[31] Bäumer T Lange R Liepert J Weiller C Siebner HR Rothwell JC. Repeated premotor rTMS leads to cumulative plastic changes of motor cortex excitability in humans. Neuroimage. (2003) 20:550–60. 10.1016/S1053-8119(03)00310-0 14527615

[32] Smith MJ Adams LF Schmidt PJ Rubinow DR Wassermann EM. Effects of ovarian hormones on human cortical excitability. Ann Neurol. (2002) 51:599–603. 10.1002/ana10180 12112106

[33] Inghilleri M Conte A Currà A Frasca V Lorenzano C Berardelli A. Ovarian hormones and cortical excitability. An rTMS study in humans. Clin Neurophysiol. (2004) 115:1063–8. 10.1016/j.clinph.2003.12003 15066531

[34] Schloemer N Lenz M Tegenthoff M Dinse HR Höffken O. Parallel modulation of intracortical excitability of somatosensory and visual cortex by the gonadal hormones estradiol and progesterone. Sci Rep. (2020) 10:22237. 10.1038/s41598-020-79389-6 33335211 PMC7747729

[35] Chagas AP Monteiro M Mazer V Baltar A Marques D Carneiro M. Cortical excitability variability: Insights into biological and behavioral characteristics of healthy individuals. J Neurol Sci. (2018) 390:172–7. 10.1016/j.jns.2018.04036 29801881

[36] Cantone M Lanza G Vinciguerra L Puglisi V Ricceri R Fisicaro F. Age, height, and sex on motor evoked potentials: translational data from a large italian cohort in a clinical environment. Front Hum Neurosci. (2019) 13:185. 10.3389/fnhum.201900185 31214003 PMC6558095

